# Solitary bone plasmacytoma of the tibia presenting as chronic osteomyelitis: A rare case report and literature review

**DOI:** 10.1097/MD.0000000000033307

**Published:** 2023-03-24

**Authors:** Xiao-Wei Yang, Gui-Chao Zhou, Zhi-Sheng Long, Fei-Peng Gong, Gang Chen

**Affiliations:** a Department of Orthopedics, Jiangxi Provincial People’s Hospital, The First Affiliated Hospital of Nanchang Medical College, Nanchang, Jiangxi, China.

**Keywords:** case reports, chronic disease, osteomyelitis, plasmacytoma

## Abstract

Plasmacytoma is a rare plasma cell dyscrasia that grows within the axial skeleton or soft tissue structures as solitary or multiple masses. The primary types are solitary plasmacytoma, including solitary bone plasmacytoma (SBP) and solitary extramedullary plasmacytoma, and multiple solitary plasmacytomas. SBP is characterized by localized proliferation of monoclonal plasma cells and is rare. However, SBP with chronic osteomyelitis is even rarer.

**Patient concerns:**

A 47-year-old man previously diagnosed with chronic osteomyelitis presented with repeated discharge and ulceration in the front of his right tibia.

**Diagnosis, interventions and outcomes:**

Lower extremity magnetic resonance imaging (MRI) and computed tomography (CT) examinations showed dead bone formation and surrounding inflammatory edema. Thus, the patient underwent dead bone excision and fenestration of the bone marrow cavity. The histopathologic examination results indicated plasmacytoma. Therefore, we administered radiotherapy with satisfactory results.

**Lessons:**

Physicians should pay close attention to chronic osteomyelitis because it may be accompanied by plasmacytoma. Postoperative pathological and immunohistochemical examinations are crucial, and surgical resection of the lesion and local radiotherapy are effective treatment methods.

## 1. Introduction

Solitary bone plasmacytoma (SBP) is rare, accounting for 2% to 5% of all plasmacytic malignancies. These lesions, which are more common in men than in women (2:1 male-to-female ratio), are characterized by local proliferation of monoclonal plasma cells, primarily in the axial skeleton, usually the vertebrae and skull. Distal adnexal bone involvement below the elbow and knee is rare, and there is no generalized involvement of myeloma-induced manifestations.^[1]^ SBP represents early-stage multiple myeloma (MM), and patients with distinct solitary lesions may have occult MM.

Given the low incidence of SBP, diagnostic criteria have been difficult to establish.^[2]^ The current guidelines provided by the International Myeloma Working Group define SBP as biopsy-confirmed plasmacytosis in bone or soft tissue in the context of normal bone marrow and skeletal examinations (except for primary isolated lesions).^[3]^ Chronic osteomyelitis is associated with avascular bone necrosis and sequestrum (dead bone) formation.^[4]^ However, osteomyelitis complicated with plasmacytoma is extremely rare.

We report the case of a 47-year-old man with SBP in the tibia that developed after 32 years of chronic osteomyelitis. To our knowledge, this is only the seventh case report of SBP associated with chronic osteomyelitis.

## 2. Case report

Thirty-two years prior at age 15, our male patient developed swelling of the medial side of his right calf with high fever but without obvious indications; he was diagnosed with acute osteomyelitis and underwent symptomatic treatment. Nearly 20 years later, the patient developed intermittent pain in the right calf, which was subsequently broken for debridement and drainage. During this time, multiple old scars were seen in the right calf. However, the patient reported no ulceration or fever during this period.

Two months ago, at age 47, the patient had calf pain again, but conventional symptomatic treatment did not relieve the symptoms. Thus, the patient was referred to our department. The patient had a high erythrocyte sedimentation rate and a high C-reactive protein level (Table 1), but the white blood cell count, alkaline phosphatase, procalcitonin, and blood cultures were normal. Therefore, a biopsy specimen of the bone and surrounding tissue was taken for culture. The culture results identified *Staphylococcus aureus* (Table 1).

**Table 1:** Serological parameters and microbiological workup.

Investigations	Measured value (Reference value)
WBC	6.15 × 10^9^/L (3.5–9.5 × 10^9^/L)
ESR	38 mm/h (0–15 mm/h)
CRP	13.20 mg/L (0–8.0 mg/L)
N%	0.688 (40%–75%)
PLT	236 × 10^9^/L (125–350 × 10^9^/L)
ALP	35 IU/L (9–50 IU/L)
PCT	0.04 ng/mL (0–0.05 ng/mL)
Biopsy specimen culture	*Staphylococcus aureus* (+)
Kappa light chain	9.95 g/L (6.29–13.5 g/L)
Lambda light chain	5.95 g/L (3.13–7.23 g/L)

ALP = alkaline phosphatase, CRP = C-reactive protein, ESR = erythrocyte sedimentation rate, N% = proportion of neutrophils, PCT = procalcitonin, PLT = platelets, WBC = white blood cells.

**Figure 1.**
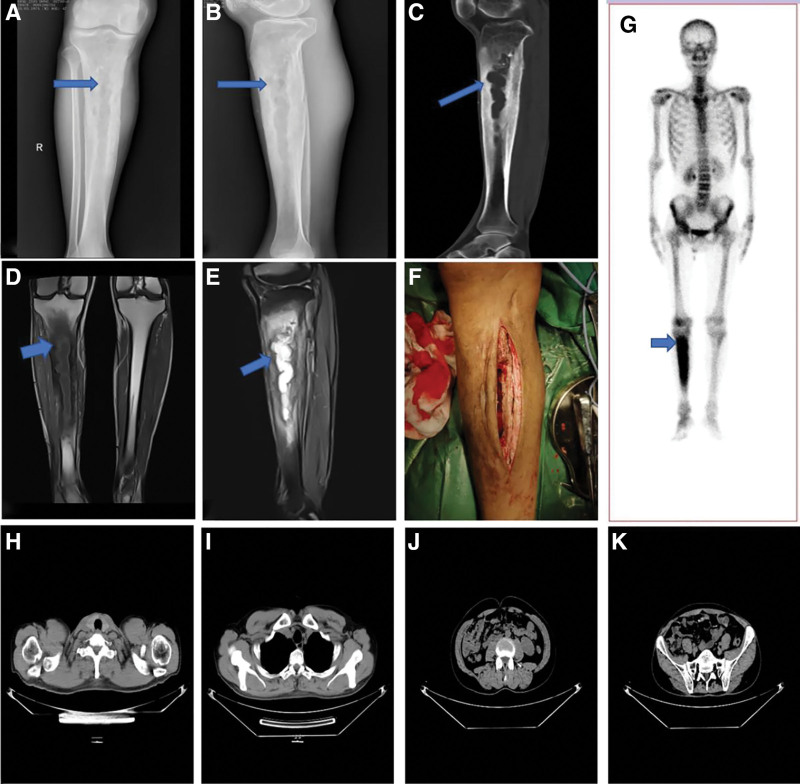
(A and B) Anteroposterior and lateral radiographic views of the proximal tibia show dead bone, an increased marrow cavity, cortical bone thickening, no periosteal reaction, and no soft tissue, representing an aggressive lesion (arrow). (C) A computed tomography (CT) scan shows an enlarged medullary cavity of the middle and upper part of the tibial shaft. Also, the normal bone trabecular structure disappeared, and the bone cortex is obviously thickened and hardened (arrow). (D) Spin echo T1-weighted magnetic resonance imaging (MRI; coronal view) of both legs (for comparison) for chronic osteomyelitis shows cortical thickening, bone-marrow edema, and a sequestrum (arrow) on the right tibia (arrow). (E) A T2-weighted MRI image shows hyperintensity and short tau inversion recovery sequences (arrow). (F) Intraoperative imaging depicts debridement, dead bone removal, and fenestration and drainage of the medullary cavity. (G) An ECT scan shows apparent, concentrated radioactivity in the upper tibia (arrow). (H–K) CT scans of the cervical vertebra (H), thoracic vertebra (I), lumbar vertebra (J), and pelvis (K) show no bone destruction and typical plasma cell tumor lesions. ECT = emission computed tomography.

A common X-ray film exam showed cortical thickening of the right tibia, bone marrow cavity structure disorder, and no periosteal reaction (Table 1). A plain computed tomography (CT) scan showed that the medullary cavity in the middle and upper part of the right tibial trunk was enlarged, the normal trabecular bone structure had disappeared, and the cortical bone was significantly thickened and hardened. A plain magnetic resonance imaging (MRI) scan showed hyperosseous hyperplasia of the right tibia (Fig. 1). Furthermore, we observed strips of irregular mixed long T1 and T2 signal shadows in the medullary cavity and strips of periosteal new bone nearby. Plasmacytomas typically have a low signal on T1-weighted MRI images (Fig. 1) and a high signal on T2-weighted and short tau reversal recovery sequences MRI images (Fig. 1).

**Figure 2.**
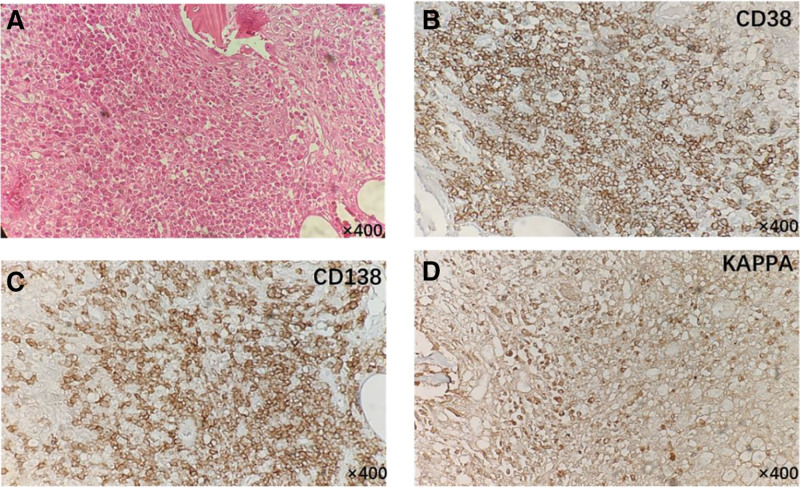
(A) Hematoxylin and eosin (H&E) staining shows monomorphic plasma cells with soccer ball chromatin with mitotic figures. (B–D) Immunohistochemistry analyses show plasma cells with CD38 positivity (B), CD138 positivity (C), and Kappa light chain restriction (D). All images are 400× magnification.

The medical history and imaging data led to a chronic osteomyelitis diagnosis. Therefore, we performed extensive debridement by creating a 10 cm^3^ cortical window to obtain adequate drainage (Fig. 1). Polymethyl methacrylate beads loaded with vancomycin were used in the medullary cavity. However, the histopathology results from the tibia (Fig. 2) suggested that the medulla contained sheets of plasmoids. Additionally, the immunohistochemistry results were as follows: cytokeratin (–), CD20 (+, less), CD3 (–), CD79a (+, less), Kappa (+), Lambda (–), CD138 (+), CD38 (+), CD56 (–), and Ki-67 (+, ~5%). Thus, the histopathological diagnosis was plasma cell myeloma. A single photon emission CT examination (Fig. 1G) depicted a more homogeneous concentration of radioactivity in the right tibia and medullary cavity. We also performed spine and pelvis CT examinations, finding no bone destruction or typical myeloma lesions (Fig. 1). After excluding MM, we diagnosed SBP based on the imaging features, plasmacytoma diagnosis, and the diagnostic criteria for solitary plasmacytoma (SP) recommended by the International Myeloma Working Group.^[5]^

Considering the patient had an infection and plasmacytoma, they received 2 weeks of intravenous antibiotics or 4 weeks of oral antibiotics postoperatively. Before stopping the antibiotic treatment, we conducted a regular blood test to confirm that the white blood cell, erythrocyte sedimentation, C-reactive protein, and procalcitonin levels were within normal ranges. After the antibiotic treatment, the patient was transferred to the oncology department for radiotherapy (40 Gy/1.8–2 Gy daily). The patient incision healed well, and they were discharged without incident. The patient had no pain or walking limitations and no sequelae or radiological signs of recurrence at the last follow-up (12 months postoperatively).

## 3. Discussion

SP is a plasma cell disorder characterized by a localized accumulation of neoplastic monoclonal plasma cells in bone or soft tissues (with no skeletal component) without evidence of systemic involvement (e.g., no clonal plasma cells in the bone marrow and no features of end-organ damage). There are 2 types of SP: SBP and solitary extramedullary plasmacytoma.^[6]^ SBP diagnoses require strict and comprehensive imaging evaluations and can only be diagnosed after excluding lesions in other body parts, which is common in patients with suspected SBP.^[7]^ Therefore, to differentiate SBP from MM, more relevant tests are necessary, including comprehensive clinical examinations, routine blood, renal function, serum calcium, and immune protein exams, as well as a bone marrow biopsy for histopathological examination. Changes in renal function and the blood calcium level, multiple bone damage sites, abnormal immune protein indexes, and an abnormal Kappa-light chain/Lambda-light chain ratio are features that help diagnose MM.^[8]^ In our case, the patient had a bone marrow lesion in the middle and upper segment of the right tibial trunk, <2% of plasma cells, and normal renal function and serum calcium distribution.

Plain and enhanced MRI scans are also helpful for differentiating SBP from MM. However, for patients with an SP diagnosis by MRI, it can reveal approximately 30% additional lesions. Positron emission tomography (PET)/CT is highly sensitive for detecting myeloma.^[9,10]^ The 2017 International Myeloma Working Group guidelines also acknowledged that PET/CT is necessary to confirm an SP diagnosis.^[5]^ Therefore, PET/CT is a valuable screening tool for myeloma lesions in SP since it examines the entire body in a single study and provides additional information to support inconclusive CT or MRI findings after treatment. Additionally, PET/CT detects disease changes faster than MRI.^[11]^ Therefore, the International Lymphoma Radiation Oncology Group recommends PET/CT as standard testing for SP. However, PET/CT exams are limited; they cannot detect very small lytic bone lesions, especially those in the skull, and they have less sensitivity for detecting diffuse changes in early bone marrow infiltration than MRI.^[6]^ In our case, the lesion was in the tibia, identified by CT, MRI, and emission computed tomography examinations.

SBP complicated with chronic osteomyelitis was first reported by Loeper in 1947^[12]^ in a 37-year-old man with a sinus tract in the femur for 27 years and plasmacytoma. Furthermore, Heilmann^[13]^ reported a case of myeloma complicating osteomyelitis. However, in both cases, the disease was widely disseminated. Next, Baitz and Kyle^[14]^ described a patient with a 40-year history of chronic osteomyelitis who also had a sinus tract in the femur and underwent several operations. The fourth case was presented by Wohlenberg,^[15]^ who further discussed SBP combined with osteomyelitis; he believed that chronic osteomyelitis, lasting 30 to 40 years, contributed to lymphoplasma cellular tissue and bone marrow becoming cancerous. Moreover, in 1984, Parsons^[16]^ described the case of a 68-year-old man with chronic osteomyelitis (40 years) diagnosed with solitary myeloma presenting as a lower femoral fracture. Finally, Roger^[17]^ reported a 42-year-old man with chronic osteomyelitis presenting with a pathological fracture in the femur; this patient suffered trauma from an accident 20 years prior, and postoperative histopathology results from the tissue around the fracture were used to diagnose SBP. To our knowledge, we are reporting the seventh case of SBP with chronic osteomyelitis (Table 2).

**Table 2:** Literature review of solitary sone plasmacytoma involving chronic osteomyelitis.

Case no.	Author	Age (yr)	Sex	Site	Osteomyelitis history	Symptoms
1	Loeper^[12]^ (1947)	30	Male	Femur	27 yr	Sinus tract
2	Heilmann^[13]^ (1957)	66	Male	Both femora	UN	Paget disease characteristics
3	Baitz^[14]^ (1964)	UN	UN	femur	40 yr	Sinus tract
4	Wohlenber^[15]^ (1970)	UN	UN	UN	UN	UN
5	Parsons^[16]^ (1984)	68	Male	Femur	40 yr	Sinus and ulcer on the lateral thigh
6	Roger^[17]^ (1992)	42	Male	Femur	20 yr	Pathology fracture

UN = unknown.

The pathogenesis of SBP, especially SBP combined with chronic osteomyelitis, remains unclear, but SBP appears to be a very rare complication of chronic osteomyelitis. The current literature and our case demonstrate that most patients have a long history of osteomyelitis, the longest of which was 40 years. Long-term inflammation may cause pathological changes in the surrounding tissues or cells, eventually leading to plasmacytoma. Wohlenberg also discussed these unusual occurrences, suggesting a similar view.^[15]^ However, several hypotheses for SBP have been proposed, such as radiation, chemical damage, viral infections, and genetic factors (e.g., deletion of chromosomes 13, 1P, 14q and the acquisition of chromosomes 19P, 9q, 1q). Additionally, the high differentiation and angiogenesis of plasmacytomas have been associated with disease progression. Interleukin-6 is a major growth factor,^[18]^ promoting B cell proliferation during bone marrow stimulation, and trauma can trigger inflammatory cytokine secretion by injured cells. Moreover, a considerable number of patients with SBP have a reported history of trauma. Some studies have investigated the association between previous trauma and SBP in younger patients. For example, 1 study investigated the association between tibial involvement and SBP in younger patients, but clear and reasonable evidence of an association was lacking. Trauma can lead to increased cytokine release, resulting in increased proliferation of plasma cells and stromal cells in bone, suggesting that trauma may be 1 disease trigger.^[19]^ However, since there are so few patients, the etiology of SBP in osteomyelitis requires further exploration.

Currently, clear and unified guidelines for treating SBP with chronic osteomyelitis do not exist. The general management of chronic osteomyelitis requires aggressive and adequate surgical debridement and long-term antibiotic therapy because local and conservative debridement has been associated with a high failure rate.^[20]^ Surgery, however, is not limited to surgical debridement; it also includes 3 ancillary aspects that contribute to successful treatment: adequate dead space management, bone stabilization, and skin graft coverage of the wound. Polymethyl methacrylate beads loaded with antibiotics have been used in the clinical treatment of chronic osteomyelitis and should be adjusted based on the culture results and other patient-specific factors.^[21]^ We identified *S aureus* in this case, which was sensitive to vancomycin. Therefore, we surgically removed the necrotic tissue and initiated drainage. Then, the medullary cavity was filled with vancomycin bone cement and treated with antibiotics for 6 weeks.

Local radiotherapy is the first choice for diagnosing solitary osteo-plasmacytoma, providing effective control.^[22]^ Ozsahin et al retrospectively assessed the outcomes of 206 patients with SBP and 52 patients with extramedullary plasmacytoma treated with radiotherapy alone (214 patients), radiotherapy combined with chemotherapy (34 patients), or surgery alone (8 patients). They found that the 5-year overall survival rate was 74%, the disease progression-free survival rate was 50%, and the local control rate was 85%. Furthermore, the local recurrence rate for patients receiving radiotherapy was 12%. In contrast, the rate was 60% for those not receiving radiotherapy.^[23]^ The optimal radiation dose for solitary osteo-plasmacytoma is unclear. Still, a previous report indicated that a radiation dose of 30 to 60 Gy was suitable,^[5]^ and the 2022 National Comprehensive Cancer Network guidelines recommended that solitary osteo-plasmacytoma be treated with radiation of the involved field at a 40 to 50 Gy/1.8 to 2.0 Gy dose.^[24]^

Surgical treatment is not the first choice for SBP; it is only recommended for unstable bone structures or nerve compression. However, radiotherapy after surgery is recommended.^[5]^ According to worldwide guidelines and clinical studies, local radiotherapy was indicated for treating SBP in our patient. Thus, we administered local radiotherapy after 4 courses of treatment (40 Gy/1.8–2.0 Gy daily). As a result, the patient incision healed well, and they were released from the hospital without incident. In addition, the patient had no pain or walking limitations, nor were there sequelae and radiological signs of recurrence at the last follow-up visit 12 months postoperatively.

## 4. Conclusions

In conclusion, SBP with chronic osteomyelitis is extremely rare, but physicians should pay close attention to patients with chronic osteomyelitis. SBP with osteomyelitis is treatable with surgical resection of the lesion, antibiotic therapy, and local radiotherapy.

## Author contributions

**Formal analysis:** Gang Chen.

**Investigation:** Fei-Peng Gong.

**Resources:** Gui-Chao Zhou, Zhi-Sheng Long, Gang Chen.

**Visualization:** Zhi-Sheng Long.

**Writing – original draft:** Xiao-Wei Yang, Gui-Chao Zhou.

**Writing – review & editing:** Zhi-Sheng Long.
